# The expanding Pandora’s toolbox of CD8^+^T cell: from transcriptional control to metabolic firing

**DOI:** 10.1186/s12967-023-04775-3

**Published:** 2023-12-11

**Authors:** Jinghong Wu, Zhendong Lu, Hong Zhao, Mingjun Lu, Qing Gao, Nanying Che, Jinghui Wang, Teng Ma

**Affiliations:** 1 Cancer Research Center, Beijing Chest Hospital, Beijing Tuberculosis and Thoracic Tumor Research Institute, Capital Medical University, Beijing, 101149 China; 2 Department of Pathology, Beijing Tuberculosis & Thoracic Tumor Research Institute, Beijing Chest Hospital, Capital Medical University, Beijing, 101149 China

**Keywords:** CD8^+^ T cells, Anti-tumor immunity, Immunotherapy

## Abstract

CD8^+^ T cells are the executor in adaptive immune response, especially in anti-tumor immunity. They are the subset immune cells that are of high plasticity and multifunction. Their development, differentiation, activation and metabolism are delicately regulated by multiple factors. Stimuli from the internal and external environment could remodel CD8^+^ T cells, and correspondingly they will also make adjustments to the microenvironmental changes. Here we describe the most updated progresses in CD8^+^ T biology from transcriptional regulation to metabolism mechanisms, and also their interactions with the microenvironment, especially in cancer and immunotherapy. The expanding landscape of CD8^+^ T cell biology and discovery of potential targets to regulate CD8^+^ T cells will provide new viewpoints for clinical immunotherapy.

## Introduction

CD8^+^ T cells, the central player of the adaptive immune system in eliminating pathogens and tumor cells, exhibit functional plasticity and complexity [1–3]. Naive CD8^+^ T cells are rapidly activated and clonal expanded to produce lots of antigen-specific effector CD8^+^ T cells and memory T cells after receiving antigens presented by dendritic cells (DCs) in peripheral lymphoid organs. The effector CD8^+^ T cells then enter the blood and migrate to the primary sites of infection or tumor, secreting cytokines such as interferon, tumor necrosis factor (TNF), and cytotoxic effector molecules such as perforin, granzyme and so on, to specifically eliminate the infected target cells or tumor cells. CD8^+^ T cells are a loyal guardian, but when CD8^+^ T cells are persistently activated or metabolically disturbed, the line of defense against pathogens and tumors will be broken. Out of control of CD8^+^ T cells are mainly manifested as exhaustion, dysfunction and ineffective monitoring, leading to immunotherapy tolerance, especially in infectious diseases and tumors [4, 5]. There have been a number of combination therapies such as anti-PD-1/PD-L1(programmed death receptor 1/programmed cell death 1 ligand 1) combined with chemotherapy, radiotherapy, angiogenesis inhibitors, agonists of the co-stimulatory molecule, stimulator of interferon genes agonists, epigenetic modulators, or metabolic modulators and so on, showing superior efficacies and higher response rates in cancer treatment [6].

In this review, we systematically describe the updates of CD8^+^ T cells development, metabolism, crosstalk with tumor microenvironment in the case of tumorigenesis. And we summarize emerging evidence that how transcription regulation and T cell metabolism will affect its ability to combat cancer. In the end, we discuss unanswered questions in the field, to gain more complete understanding of T cells and provide new ideas for future CD8^+^ T cell-based therapies.

### Transcriptional mechanisms in CD8^+^ T cell differentiation

#### CD8^+^ T cell activation

**Fig. 1:**
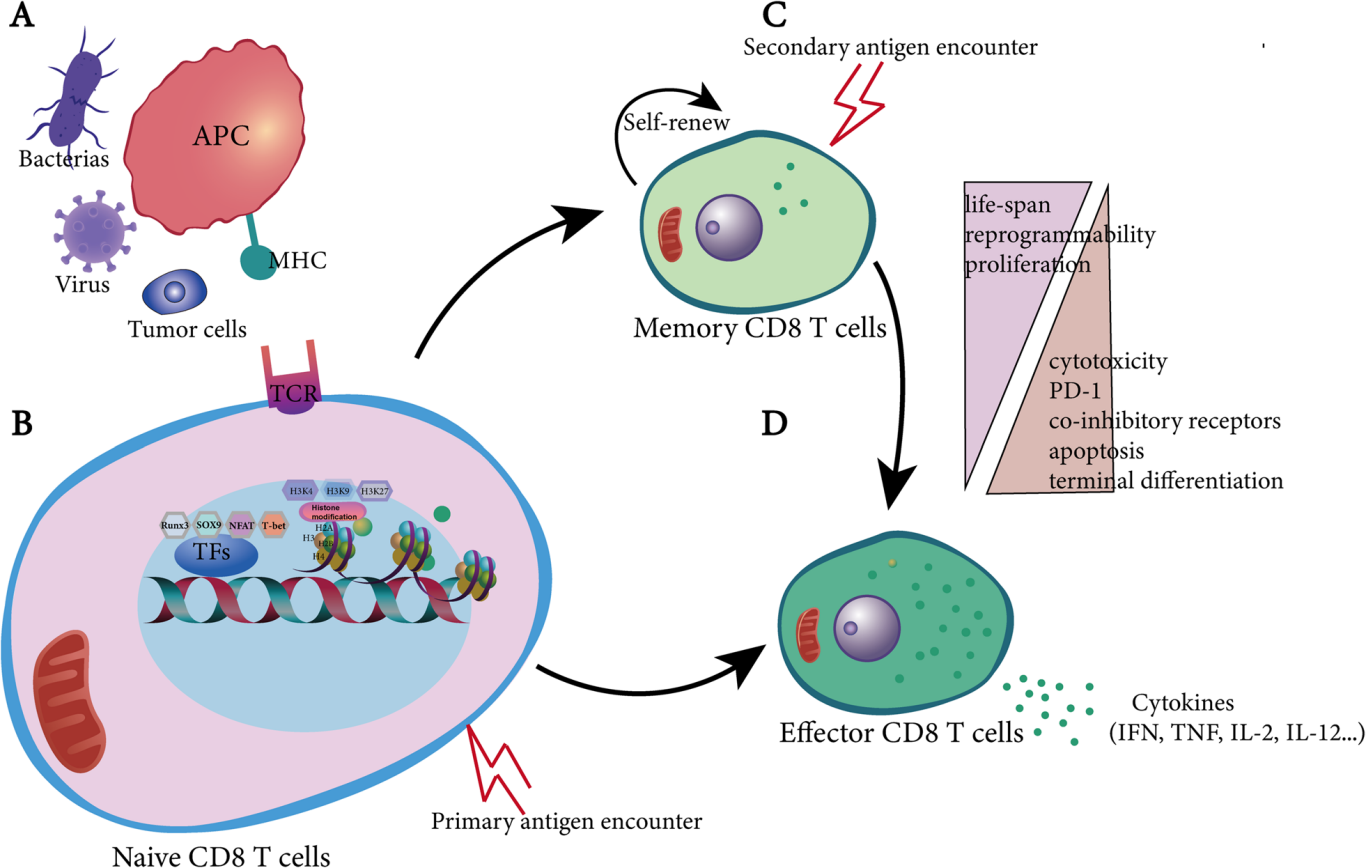
Transcriptional and epigenetic mechanisms involved in CD8^+^ T cell development. **A** APCs can recognize and present the antigens, and then present to CD8^ +^ T cells by MHC, CD8^ +^ T cells are activated. **B** When naive CD8^+^ T cells activated, most of them transform into effect CD8^+^ T cells and a small part transform into memory CD8^+^ T cells. Naive CD8^+^ T cells translate effector CD8^+^ T cells, this process is regulated by transcription factors, such as T-bet, and so on, at the same time, epigenetics is changed. C: memory CD8^+^ T cells can self-renew, and when it’s stimulated by second antigens, memory CD8^+^ T cells rapidly transform into effect CD8^+^ T cells. D: effect CD8^+^ T cells secrete cytokines such as TNFs in response to stimulation. *APC* antigen presenting cell; *MHC* major histocompatibility complex, *TFs* transcription factors, *PD-1* programmed death receptor 1

CD8^+^ T cells are important in adaptive immunity to tumor. Activation of naive CD8^+^ T cells trigger the change of cell cycle, protein expression, metabolism, and generation of distinct cellular phenotypes [7]. Once activation, naive T cells could differentiate into short-lived or terminal effector cells (SLECs/TEs) as well as long-lived memory precursors (MPs), and switch from quiet to active state that enable amplification up to 15–20 times approximately within one week and increase up to 50,000-fold in cell number [8–10]. The variety, strength and duration of antigen are key determinants of T cell differentiation [11, 12]. At the same time, APCs and/or CD4^+^ T cells secret co-stimulatory signals and cytokines that influence CD8^+^ T cells differentiation. Then CD8^+^ T cells undergo differentiation and expansion to generate a great numbers of effector cells which are able to migrate into the periphery. Mechanistically, naive CD8^+^ T cells are activated by recognition of specific peptides presented by major histocompatibility class I (MHC-I) on antigen presenting cells (APCs) in peripheral lymphatic organs (Fig. 1), however, tumor cells can significantly reduce MHC-I antigen presentation, thereby "hiding" in front of CD8^+^ T cells and achieving immune escape. This type of tumor with T cell infiltration and weak immune response is also known as a "cold tumor" [13]. Wang’s latest research indicate that SUSD6 and TMEM127 are two membrane molecules that simultaneously interact directly with MHC-I and jointly recruit WWP2 to form a quaternary complex. In the presence of SUSD6 and TMEM127, WWP2 mediates MHC-I ubiquitination as well as lysosomal degradation, thereby reducing MHC-I surface expression, loss of SUSD6 enhances MHC-I surface expression, which can promote the function of CD8^+^ T cells, thereby enhancing tumor immune surveillance [14]. In addition to MHC-I, MHC-II are also important for T cell differentiation. Research from Booki Min reported that MHC-II^−/−^ CD8^+^ T cells are hyperproliferated under lymphopenic conditions, differentiated into effector cells producing proinflammatory cytokines, and mediated more severe tissue inflammation compared with wide type CD8^+^ T cells, the reason is that, as a MHC-II ligand, LAG3 is markedly enhanced in MHC-II^−/−^ CD8^+^ T and blockade of MHC-II-LAG3 interaction further promote T cell expansion [15].

#### CD8^+^ T cell differentiation

CD8^+^ T cell differentiation accompanies with its activation. In this part, we will discuss the role of transcription factors, T-bet, Eomesodermin (Eomes) and Runx3 in the differentiation of CD8^+^ T cell.

As a member of the T-box family, T-bet is the main regulator of type I differentiation in CD8^+^ T cells and is necessary for the expression of IFN. CD8^+^ T cells expressing T-bet represent short-lived effector differentiation and associates with the KLRG1^hi^ and CD127^lo^ phenotype [16–18]. Eomes is a different transcriptional factor which belongs to T-box family. Eomes  expression increase from the effector to memory phases of a CD8 T cell response while T-bet expression is observed to be maximal during effector phase [19, 20]. IL-12 upregulates T-bet expression but represses its consistent transcription, which is consistent with IL-12’s effect in regulation of robust, short-lived effector cells [21]. T-bet, also has long been known to be a key transcription factor for effector and memory CD8^+^ T cells (Fig. 1B) [17, 22, 23]. T-bet regulates CD8^+^ T cell effector and memory differentiation, enhances the expression of IL-2R and also helpful for cytotoxic CD8^+^ T cells to secrete IFN-γ, perforin and granzyme B [19, 20, 22]. Reiner et al. reported that T-bet and Eomes deficiency fail to respond to lymphocytic choriomeningitis virus (LCMV) infection [24]. T-bet maintains a steady state and delet of T-bet-expressing Treg cells results in severe Th1 autoimmunity in mouse [25]. IL-12 modulates T-bet in a dose-dependent manner and high amounts of T-bet induced KLRG1^hi^ IL-7R^lo^ short-lived effector cells, but lower amounts upregulated the development of KLRG1^lo^ IL-7R^hi^ memory precursor effector cells [18]. Meanwhile, research revealed that T-bet and Eomes regulate CD8^+^ T cell exhaustion which correlate with the T-bet nuclear localization, and from Chen’s research, we know that TCF-1 participates the T-bet-to-Eomes transcription factor transition in progenitor exhausted CD8^+^ T cells by upregulating Eomes expression and driving c-Myb expression that controlled Bcl-2 and survival [26, 27]. Unexpectedly, Iwata et al. found that T-bet also acts as a repressor of Type I interferons (IFN-I) transcription factors and IFN-I stimulated genes in Th1 cell that restrains Th1 response [28]. These findings suggests that T-bet is necessary to determine CD8^+^ T cell fate and its function is related to the localization of the cell.

Eomes is highly homologous to T-bet and expressed in activated CD8^+^ T cells and in activated NK cells. It has cooperative functions with T-bet in CD8^+^ T cells [29]. Andrew et al. cross-bred T-bet^−/−^ mice with Eomes^−/−^ mice to obtain dual gene knockout mice. The mice showed a decrease in the proportion of CD8^+^ T cells and the production of IFN-γ, and the cytotoxic activity under the infection with LCMV [30]. T-bet and Eomes are also involved in regulating the differentiation of CD8^+^ T cells into effector T cells and memory T cells. Laura et al. used flow cytometry to detect the expression levels of T-bet and Eomes on immature CD8^+^ T cells, central memory CD8^+^ T cells, effector memory T cells, and effector CD8^+^ T cells. The results showed that T-bet had the highest expression level on effector CD8^+^ T cells, Eomes has the highest expression level on effector memory T cells [31–34]. Other group also confirmed the regulatory effect of T-bet and Eomes on memory T cells [35]. Banerjee et al. found that Eomes knockout mice also had defects in the formation of long-term memory T cells, cell stability, and cell renewal ability [36]. Therefore, the differentiation direction of CD8^+^ T cells depends on the expression levels of T-bet and Eomes. TGF-β (transforming growth factor β) and Eomes signal coordinate to promote the homeostasis of CD8^+^ Treg cells. Simultaneous disruption of both TGF-β receptor and Eomes in T cells result in lethal autoimmunity [37]. Ectopic expression of Eomes is sufficient to activate effector CD8^+^ T cells that secrete IFN-γ, perforin and granzyme B [20]. Eomes-dependent loss of CD226 related to tumor-infiltrating lymphocytes (TILs) with reduced anti-tumor functions [38]. All in all, the regulation for CD8^+^ T cells is multifactors coordination in different states and periods.

In addition, Runx3 can be considered as another transcription factor. Runx3 is an important regulator of T_RM_ cell differentiation and homeostasis through TGF-β dependent transcriptional mechanism [39]. Runx3 and T-bet colocalization with Batf that mediated effector CD8^+^ T cell differentiation [40]. Runx3 can be regarded as a tumor suppressor transcription factor which delays melanoma growth, mortality and enhanced tumor specific CD8^+^ T cell abundance [41]. Through computational biology and RNA interference screening techniques, Goldrath et al. found that Runx3 is a key regulatory factor involved in T_RM_ differentiation and homeostasis in various tissues, and can participate in special gene expression programs in CD8^+^ T cells infiltrating normal tissues and tumors. It also has been confirmed through a melanoma mouse model that overexpressing Runx3 in T cells can slow down tumor growth and prolong survival, while the absence of Runx3 leads to worse outcomes [41]. Regulating the activity of Runx3 in T cells can affect the accumulation of T cells in solid tumors, which may help researchers improve current cancer immunotherapy. In the future, we can use Runx3 to reprogram CD8^+^ T cells, thereby driving their killing effect in tumors. Green et al*.* used CRISPR-based screen to identify the mammalian BRG1/BRM-associated factor (cBAF), which are positively correlated with the differentiation of activated CD8^+^ T into effector cells and negatively correlated with memory T cell formation [42]. Whether there is a synergistic relationship between T-bet and BRG1/BRM-associated factor has not been studied.

In conclusion, Eomes, Runx3 and T-bet are members of an interactional transcriptional network necessary for CD8^+^ T cell differentiation program and acquisition of effector functions. T cell receptor (TCR) signal activates T-bet that promotes IFN-γ expression. Runx3 induces the expression of IFN-γ and upregulates granzyme B, then Runx3 induces Eomes and subsequent the expression of perforin and IFN-γ expression [43]. The differential expression and function of these factors during effector and memory stages suggest an important role for them in the induction and maintenance of genetic programs that regulate effector and memory CD8^+^ T cell differentiation and imply that its use may greatly benefit tumor therapy. There are also other transcription factors that regulate CD8^+^ T cell that it is not mentioned in the text (Table 1).

**Table1:** Transcription factors associated with CD8^+^ T cells

TFs	Function	Refs.
T-bet	Affect CD8^+^ T cells fate; promote INF-γ expression; depress IL-17 production; interaction with mTOR and IL-12	[17, 18, 22–24, 156]
Runx3	CTL proliferation; granzyme expression; interaction with T-bet and Eomes;	[43, 157]
Sox9	Negatively regulated CD8^+^ T cells	[158]
NFAT	Inhibition the production of cytokine	[159]
Eomes	Affect CD8^ +^ T cells fate; the homeostasis of CD8^+^ T cells; IFN-α production; cytotoxicity; granzyme and perforin production; repress IL-17 and IL-12; interaction with mTOR	[19, 21, 34, 43, 156, 160]
c-Myc	The homeostatic proliferation of memory CD8^+^ T cells	[161]
Blimp-1	The homeostasis of CD8^+^ T cells; cytotoxicity; interaction with IL-2	[162, 163]
Bcl-6	The generation and maintenance of memory CD8^+^ T cells	[164]
NF-κB	The generation and maintenance of memory CD8 ^+ ^T cells; cytokine production	[165]
Notch	CD8^+^ T cells proliferation; INF-γ expression; expression of eomesodermin, perforin, and granzyme B	[166, 167]
STAT1/4	Cytotoxicity generation and promote INF-γ expression	[6, 168, 169]

### Epigenetic regulation of CD8^+^ T cell

Growing studies have shown that epigenetic mechanisms cooperate with transcription factors, which is crucial for the transcriptional changes associated with CD8^+^ T cell differentiation (Fig. 1B). Histone post-translational modifications and DNA methylation are the main epigenetic mechanisms. DNA methylation mainly occurs on CG dinucleotide (CpG)-dense regions, namely CpG islands which are located at transcriptional start sites and associate with transcriptional repression [44, 45]. Understanding epigenetic mechanisms that regulate the differentiation of CD8^+^ T cell would have implications for both T cell biology and immunotherapy. Asymmetric expression and directed activity of epigenetic modifying proteins during CD8^+^ T cell differentiation regulate subset-specific cellular functions and may even be involved in fate decisions during the early stages of naive T cell activation. Knockout of the gene encoding methyl-CpG-binding domain protein 2 (MBD2) leads to differentiation defects in CD8^+^ T cells [2, 46, 47]. As a component of the H3K27me3 reader complex PRC1, the expression of BMI1 is regulated by TCR in both naive CD8^+^ T cells and memory precursor T cells, and BMI1 participates in cellular senescence and apoptosis through regulation of the gene expression of p16INK4A and p14ARF, however, it disappears in terminally differentiated effector T cells [48–51]. Similar results are also observed in histone-lysine N-methyltransferase, EZH2, which belongs to part of the H3K27me3 writer complex PRC2. EZH2^+^CD8^+^ T cells increased the polyfunctionality and resistance to spontaneous and induced apoptosis, which is regulated by Notch pathway [52]. At the same time, to characterize of the proteins that participate the transcriptional effects of DNA methylation in CD8^+^ T cells needs to be further clarified.

The ability to modulate the function of T cells through epigenetic regulation has important therapeutic implications. As a reader of acetylated lysines, BRD4, and the histone deacetylase sirtuin 1 (SIRT1), can be inhibit by JQ1 that is a pharmacological inhibitor of the BET family of bromodomain-containing proteins. Mechanistically, JQ1 reduces BATF expression, increases proliferation and cytokine production of CD8^+^ T [53, 54]. There are more epigenetic regulators that participat in the differentiation and function of CD8^+^ T cell, which need more exploration.

### Mechanisms of CD8^+^ T cells metabolic regulation

It is universally accepted that the functions of T cells activation, differentiation and effector are basic in T cell biology, which are closely related to changes in the cellular metabolic programs. Metabolic pathways such as glycolysis, fatty acid synthesis and mitochondrial metabolism play significant roles in T cell immunometabolism [28]. In healthy persons, metabolically quiescent T cells reside in lymph nodes and peripheral tissues in order to recognize antigens. Once infection, T cells are activated in a specific manner to become effector T cells such as proliferate and/or differentiate which are accompanied by important changes in cellular metabolism, and this progress can be defined as metabolic reprogramming [5]. At the same time, the shift in energy production is accompanied by mitochondrial ultrastructural modifications that facilitate the metabolic transition [55]. Hypoxia-induced mitochondrial remodeling can also promote T cell exhaustion, reducing antitumor immunity. Up to date, metabolic pathways have been manipulated to treat immune-dysregulatory diseases. One of the therapies is rapamycin (sirolimus) that targets PI3K/Akt and glucose transporter 1(GLUT1) to regulate mTOR under the stimulation of TCR. Leucine, glutamine, and arginine also regulate mTOR expression; in patients with atopy due to CARD11 loss-of-function (LOF). Glutamine supplementation can promote Th1 differentiation through mTOR, rescuing the atopic T cell phenotype [56]. The metabolic profile of T cells is complexly linked to their differentiation state and have a considerable impact on the generation and duration of effector T cell activity [57]. Here, we discuss in detail the metabolic states of CD8^+^ T cells providing a guide of therapeutic basis for cancers.

#### CD8^+^ T cell metabolism from quiescence to activation

T cell metabolism is indispensable not only for priming cells for rapid activation,

but also maintaining homeostasis in naive and memory cells. Metabolic progress regulates T cell quiescence. Naive T cells have lower mitochondrial activity and glucose uptake, and produce ATP through mitochondrial OXPHOS29 to support T cell homeostasis which is different from antigen-stimulated T cells [58–60]. At the same time, T cell metabolism promotes and on behalf of the activation and differentiation and modifies gene transcription and post-transcriptional regulation [61, 62]. Naive T cells enter peripheral tissue from thymus and are actively maintained during cell cycle by combining TCR/CD3 and stimulated by IL-7 [63]. Activation of T cells show clonal expansion, cell growth and differentiation which is mediated by cell surface receptors, oxygen levels and nutrient availability [64]. The level of acute T cell activation relies on the co-stimulation receptors such as CD3, CD4, CD8, CTLA4 etc. and the activation of TCR [65]. TCR recognizes CD4/CD8 co-receptors and MHC-peptide complex that upregulate lymphocyte-specific protein kinase (Lck) and protein tyrosine kinase (PTK) C-terminal Src kinase (Csk), then induces the activation, recruitment and phosphorylation of the zeta-chain associated protein kinase 70 (ZAP70). ZAP70 upregulates phospholipase Cg1 (PLCg1), which stimulates calcium mobilization, activates protein kinase C (PKC) and Ras pathway [66–68]. When naive T cells transform to activated T cells, the metabolic activity is from low to high [69, 70]. Activated T cells rely on nutrient uptake. AMP-dependent protein kinase (AMPK), as the energy sensor in T cells can be activated by calcium calmodulin dependent protein kinase 2 (CaMKK2) and low levels of ATP and liver kinase B1 (LKB1)-dependent phosphorylation [71–73]. Meanwhile, TCR/CD3-CD28 signaling participated in mitochondrial biogenesis, which prepared for T cell proliferation and growth. Activated TCR/CD3-CD28 signaling phosphorylated PI3K-Akt -mTOR1/2 pathway, too, which subsequently regulated the important upstream regulator GTPase, tuberous sclerosis complex 2 (TSC2) [72, 74].

**Fig. 2:**
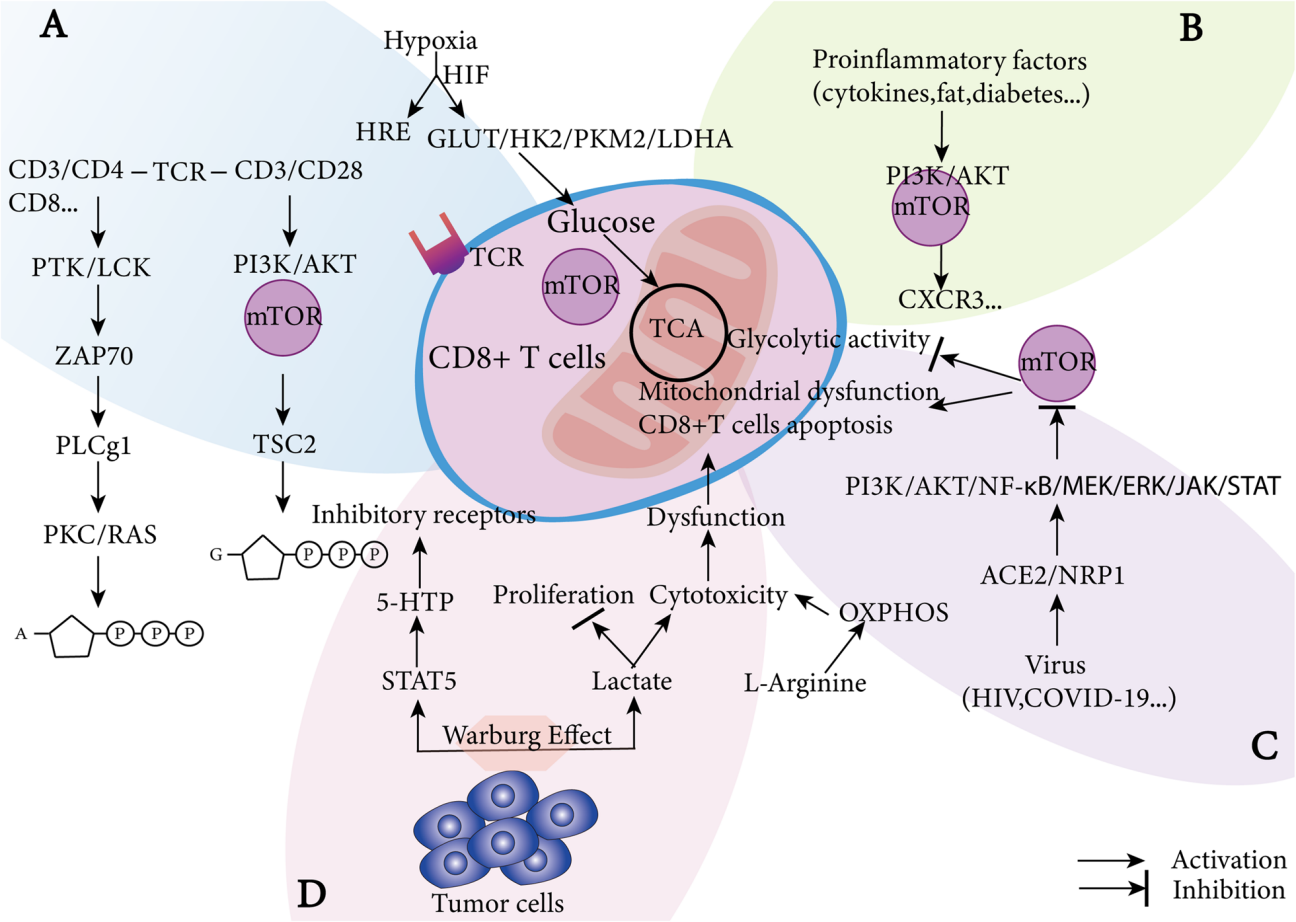
CD8^+^ T cell metabolism. **A** normal CD8^+^ T cells metabolism. CD8^+^ T cell metabolism regulated by lots of kinases, eventually producing ATP and GTP. **B** CD8^+^ T cells metabolism in an inflammatory environment (fat, diabetes…). Proinflammatory factors regulate CD8^+^ T cells metabolism through the PI3K/AKT/mTOR pathway and induce CD8^+^ T cells to produce CXCR3, then CD8^ +^ T cells dysfunction. **C** CD8^+^ T cells metabolism in virus environment. The virus enters CD8 + T cells through the ACE/NRP receptors, alters the metabolism of CD8 + T cells, and causes apoptosis. **D** Tumor cells and CD8^+^ T cells compete for body nutrients, in addition, harmful substances are released from tumor cells, which cause dysfunction and exhaustion of CD8^+^ T cells. *HRE* hypoxia response elements, *GLUT* glucose transporter, *mTOR* mammalian target of rapamycin, *TCA* tricarboxylic acid cycle, *5-HTTP* 5-hydroxytryptophan, *TCR* T cell receptor

In addition to nutrient availability and cell surface receptors, oxygen tension is also necessary for T cell metabolism. T cells are mobile through obtaining a suitable aerobic environment in the body. Naive T cells are in a low oxygen environment and activated T cells are exposed in high oxygen levels in the arterial blood and lung as well as the hypoxic conditions in tumors and inflammation sites [75–77]. The state of T cells is influenced by exposure to hypoxia that mainly mediated by HIF-1α, which translocates into the nucleus to bind hypoxia response elements (HREs) [78, 79]. HIF-1α mediates metabolic shift by regulating the expression of genes include GLUT1, HK2, PKM2, LDHA (Fig. 2A) [79–81]. Different metabolic programs define different T cell subsets, and T cells state can be manipulated via modulating metabolic activity. However, the mechanism of how metabolism influences the function of T cell in response to infections and tumors are not fully understood and whether the normal functions of T cells can be restored by regulating metabolism needs to be further elucidated.

#### CD8^+^ T cell metabolism in cancer

**Fig. 3:**
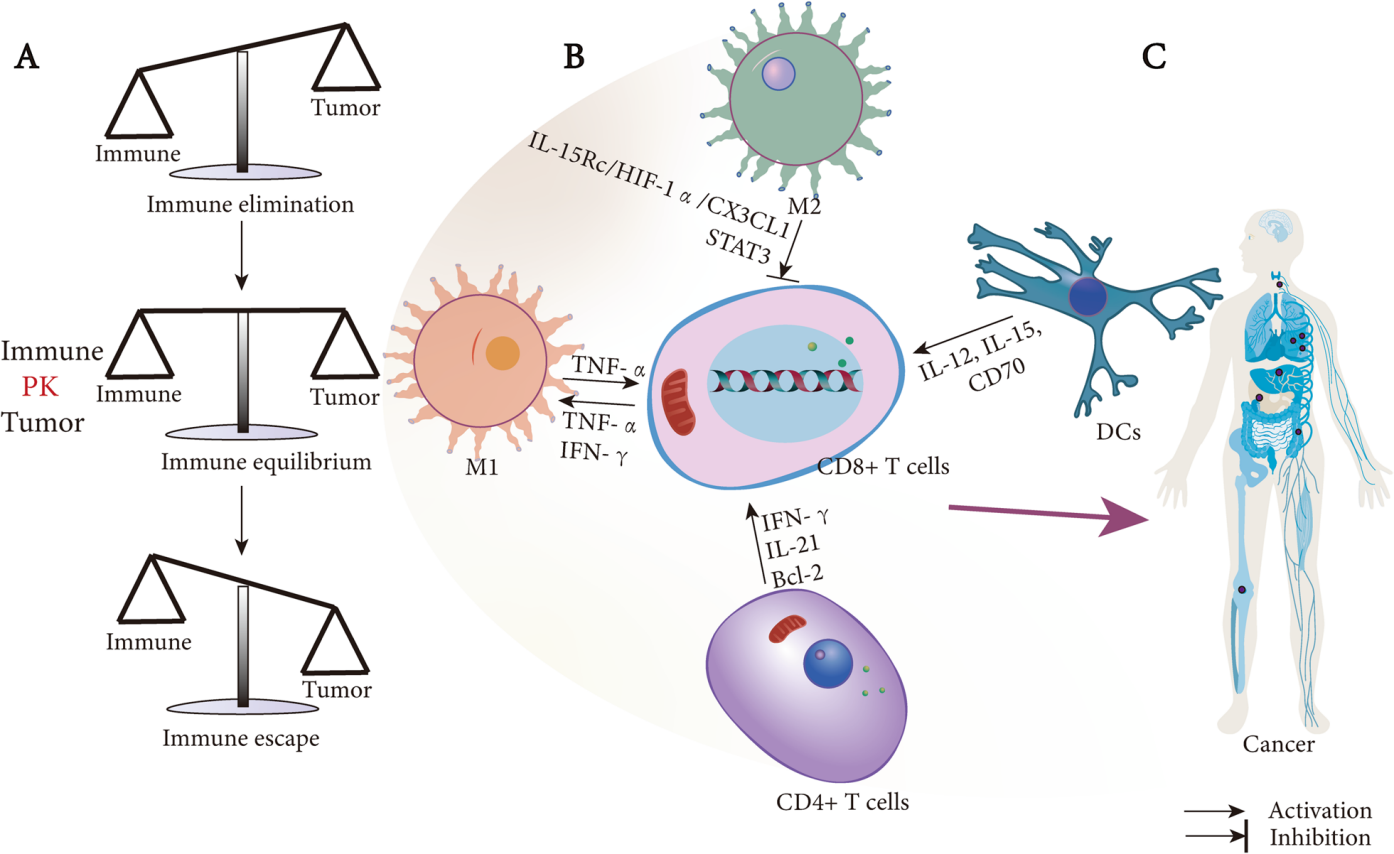
CD8^+^ T cell interaction with other immune cells in tumor microenvironment. **A** the check and balance between immune system and tumors. The three phases between immune system and tumors, elimination, equilibrium and escape, respectively. **B** CD8^+^ T cell in TME. There are a variety of cells and cytokines in TME, that crosstalk with each other to affect the function of CD8^+^ T cells, and M1 is tumor-suppressing, M2 is tumor-promoted. CD4^ +^ T cell and DCs are stimulators for CD8^+^ T cells. **C** when immune system is out of control and defeated, then cancer outgrowth. *M1* M1 type macrophages, *M2* M2 type macrophages, *DC* dendritic cell, TME tumor microenvironment

There is a bidirectional relationship between the occurrence and development of tumors and tumor microenvironment (TME) where include stromal, endothelial, immune, tumor cells, cytokines and chemokines (Figs. 2D and 3) [82]. Accumulating evidences clarify that the regulation of metabolism in TME are associated with the function of T cell and tumor cells, and play a main role in shaping anticancer immune responses [83]. Tumor cells compete with T cells for nutrients to meet their needs for proliferation and migration, under aerobic conditions. Tumor cells preferentially utilize glycolysis and metabolize approximately tenfold more glucose to lactate than normal tissues, this phenomenon is defined Warburg effect (Fig. 2D) [84]. We will discuss glucose and amino acids metabolism in next.

Glucose participates in T cell proliferation, function and regulates cell fate. Both effector T cells and hyperactive cancer cells are heavily dependent on glucose metabolism. Glycolysis is important for sustaining effector T cell immune function such as the secretion of IFN-γ. Glucose deprivation selectively inhibited the production of IFN-γ, granzyme B protein, cyclin D2 protein, cytolytic activity [82, 85]. In some glycolytic tumors, CD8^+^ T cell proliferation and infiltration are very low, which associate with tumor cells that limit the energy metabolism of CD8^+^ T cells. As the glycolysis enzyme, GAPDH binds the AU-rich region in the 3′ untranslated regions of cytokine messenger RNAs and downregulate the expression of protein [86–88]. T cell function is impaired through mTOR under low levels of glucose, and the transcriptional level of IFN-γ is diminished under the background of lowed activity of mTOR and phosphorylation of the ribosomal protein S6 kinase beta-1 (p70S6K) [89].

In addition to glucose, the accumulation of the glycolytic product lactate is negative for effector T cell function and antitumor effect, and lactate impairs CD8^+^ T cell and NK-cell infiltration and activity in melanoma [90]. However, CD8^+^ T cell seems smart and flexible in such hostile environment. CD8^+^ T cells upregulate the catabolism of fatty acid so that provide energy for preserving effector function in TME and the activation of peroxisome proliferator receptors is positive for T cell function and delays tumor growth [91]. Additionally, acetate can rescue IFN-γ production via upregulating chromatin accessibility and histone acetylation in glucose-limited T cells [92]. Thus, some metabolic targets are potential to rescue CD8^+^ T cell function in a hostile environment.

Except glucose metabolism, amino acids are indispensable for T cell function and differentiation, too. Green and Frauwirth reported that glutamine as an important source for active T cells was regulated by ERK/MAPK [93, 94]. Glutaminase deficiency abolished T cell activation and Th17 differentiation, but promoted CD4^+^ Th1 and CD8^+^ CTL cells differentiation and effector function via T-bet [95]. In addition, dynamic proteomic and metabolomic analysis identified that l-arginine is a key metabolite which promotes OXPHOS, boosts T cell survival and generates antitumor memory-like T cells [96]. Under the persistent stimulation of IL-2, STAT5 is activated and then induces strong expression of tryptophan hydroxylase 1, thus catalyze the conversion to tryptophan to 5-hydroxytryptophan (5-HTP), which upregulate inhibitory receptors expression, thereby rendering CD8^+.^ T cells dysfunctional in the TME [97]. Tumor cells methionine consumption is an immune evasion mechanism. Reducing methyl donor S-adenosylmethionine (SAM) and methionine result in loss of dimethylation at lysine 79 of histone H3 (H3K79me2), which led to decreased expression of STAT5 and impaired CD8^ +^ T cell immunity [98].

The specific impact of glucose and amino acid on CD8^+^ T cells fate and function thus suggest the possibility of immunomodulation within the TME via the manipulation of glucose and amino acid levels. The balance of this competition has been linked to the activity of metabolic enzymes, and metabolic enzymes could be a potential and effective target for immune therapy. While methotrexate is the oldest but still one of the most effective available chemotherapeutic treatments in clinical, it’s necessary to explore more antitumor drugs that target metabolism so as to improve the outcome from clinical treatment [99, 100]. At the same time, metabolism treatment combined with immune treatment are expected to lead to novel and highly specific targets. As it shown in Tables 2 and 3, the clinical trials involving CD8^+^ T cells are concluded.

**Table 2:** Anti-PD-1/PD-L1 combinational therapy and applications

anti-PD-1/PD-L1	combinational therapy	cancer	Refs.
Pembrolizumab	Pemetrexed; carboplatin; paclitaxel; gemcitabine; cisplatin; 5-fuorouracil; trastuzumab; radiotherapy; Axitinib; Lenvatinib; PF-05082566	Solid tumors; NSCLC; SCLC; TNBC; GC;	[170–173]
Nivolumab	5-fuorouracil; oxaliplatin; capecitabine; oxaliplatin; ipilimumab; radiotherapy	NSCLC; SCLC; GC; colorectal cancer; Esophageal adenocarcinoma	[174, 175]
Camrelizumab	Carboplatin; pemetrexed; gemcitabine; cisplatin; apatinib	Nasopharyngeal carcinoma; NSCLC; GC; HCC; SCLC; esophageal squamous cell carcinoma	[176, 177]
Tislelizumab	Paclitaxel; carboplatin; platinum; pemetrexed; pamiparib	NSCLC	[178–180]
Atezolizumab	Bevacizumab; paclitaxel; carboplatin; etoposide; KY1044; alectinib; ipatasertib	NSCLC; SCLC; TNBC; HCC	[181–183]
Durvalumab	Etoposide; carboplatin; cisplatin; axitinib; radiotherapy	NSCLC; SCLC; RCC	[184–187]

Abbreviations: NSCLC: non-small cell lung cancer; SCLC: small cell lung cancer; RCC: renal cell carcinoma; TNBC: triple-negative breast cancer; HCC: hepatocellular carcinoma; GC: gastric cancer

**Table 3:** Clinical trials related to CD8^+^ T cells

NCT number	Study title	Study status	Interventions
NCT03093688	Clinical Safty and Efficacy Study of Infusion of iNKT Cells and CD8^+^ T Cells in Patients With Advanced Solid Tumor	ACTIVE_NOT_RECRUITING	BIOLOGICAL: Infusion of iNKT cells and CD8^+^ T cells
NCT02424916	Adoptive Transfer of Specific Melanoma Antigens CD8^+^ T Cells in Metastatic Melanoma Patients: a Phase I/II Study	COMPLETED	BIOLOGICAL: Melanoma antigens-specific CD8^+^ T lymphocytes
NCT04965649	Is There an Association Between Innate CD8^+^ T Cells and the Evolution of Tyrosine Kinase Inhibitor Resistance Mutations in Phi^ +^ Hematological Malignancies	RECRUITING	GENETIC: Phenotyping of total and innate CD8 + T cells by flow cytometry
NCT03175705	Adoptive Transfer of Specific HCC Antigens CD8^+^ T Cells for Treating Patients With Relapsed/Advanced HCC	UNKNOWN	BIOLOGICAL: HCC antigens-specific CD8^+^ T lymphocytes|DRUG: IL-2|DRUG: Tegafur
NCT05902520	Adoptive Cell Therapy Using Cancer Specific CD8^ +^ Tumor Infiltrating Lymphocytes in Adult Patients With Solid Tumors	RECRUITING	BIOLOGICAL: DP CD8 TIL|BIOLOGICAL: DP CD8 TIL KD|BIOLOGICAL: Low dose IL-2
NCT03068624	Autologous CD8^+^ SLC45A2-Specific T Lymphocytes With Cyclophosphamide, Aldesleukin, and Ipilimumab in Treating Patients With Metastatic Uveal Melanoma	ACTIVE_NOT_RECRUITING	BIOLOGICAL: Aldesleukin|BIOLOGICAL: Autologous CD8^+^ SLC45A2-specific T Lymphocytes|DRUG: Cyclophosphamide|BIOLOGICAL: Ipilimumab
NCT04713046	Safety and Efficacy of Allogeneic HPV-specific T Cells in Adults With Recurrent or Metastatic HPV16^ +^ Cancers	RECRUITING	BIOLOGICAL: CD8 reduced peripheral blood cells taken from related donors vaccinated against HPV16|BIOLOGICAL: Non-myeloablative allogeneic bone marrow transplant from related donors vaccinated against HPV16
NCT02027935	CD8^+^ Antigen-Specific T Cells, Cyclophosphamide, Aldesleukin, and Ipilimumab in Treating Patients With Metastatic Melanoma	ACTIVE_NOT_RECRUITING	BIOLOGICAL: Aldesleukin|BIOLOGICAL: Autologous CD8 ^+ ^Melanoma Specific T Cells|DRUG: Cyclophosphamide|BIOLOGICAL: Ipilimumab|OTHER: Laboratory Biomarker Analysis
NCT03450122	Modified T Cells, Chemotherapy, and Aldesleukin With or Without LV305 and CMB305 in Treating Participants With Advanced or Recurrent Sarcoma	COMPLETED	BIOLOGICAL: Aldesleukin|BIOLOGICAL: Autologous NY-ESO-1-specific CD8-positive T Lymphocytes|DRUG: Cyclophosphamide|BIOLOGICAL: Dendritic Cell-targeting Lentiviral Vector ID-LV305
NCT01513408	Relevance of T Lymphocytes Tumor Infiltrates CD8 and Foxp3 as Immune Prognostic Biomarker in Breast Cancer Treated by Neo Adjuvant Chemotherapy	ACTIVE_NOT_RECRUITING	OTHER: immunohistochemical detection of lymphocytes T CD8^+^ /Foxp3 ratio
NCT05430555	A Phase 1/2, First-in-Human, Open-Label, Accelerated-Titration, Two-Part Clinical Trial of TK-8001 in Patients With HLA-A*02:01 Genotype and Advanced-Stage/Metastatic MAGE-A1 + Solid Tumors	RECRUITING	BIOLOGICAL: Autologous CD8^+^ T-cells, transduced with MAGE-A1 directed TCR
NCT03338972	Immunotherapy With BCMA CAR-T Cells in Treating Patients With BCMA Positive Relapsed or Refractory Multiple Myeloma	COMPLETED	BIOLOGICAL: Autologous Anti-BCMA-CAR-expressing CD4^+^ /CD8^+^ T-lymphocytes FCARH143|DRUG: Cyclophosphamide|DRUG: Fludarabine|PROCEDURE: Leukapheresis
NCT03747484	Gene-Modified Immune Cells (FH-MCVA2TCR) in Treating Patients With Metastatic or Unresectable Merkel Cell Cancer	RECRUITING	BIOLOGICAL: Autologous MCPyV-specific HLA-A02-restricted TCR-transduced CD4^+^ and CD8^+^ T-cells FH-MCVA2TCR|DRUG: Avelumab|BIOLOGICAL: Pembrolizumab|BIOLOGICAL: Interferon Gamma-1b
NCT02319824	NY-ESO-1-Specific T-cells in Treating Patients With Advanced NY-ESO-1-Expressing Sarcomas Receiving Palliative Radiation Therapy	COMPLETED	BIOLOGICAL: Autologous NY-ESO-1-specific CD8-positive T Lymphocytes|OTHER: Laboratory Biomarker Analysis|RADIATION: Palliative Radiation Therapy
NCT03103971	huJCAR014 CAR-T Cells in Treating Adult Patients With Relapsed or Refractory B-Cell Non-Hodgkin Lymphoma or Acute Lymphoblastic Leukemia	ACTIVE_NOT_RECRUITING	BIOLOGICAL: Autologous Human Anti-CD19CAR-4-1BB-CD3zeta-EGFRt-expressing CD4^+^ /CD8^+^ T-lymphocytes|DRUG: Cyclophosphamide|DRUG: Fludarabine|OTHER: Laboratory Biomarker Analysis|PROCEDURE: Leukapheresis|OTHER: Pharmacological Study

To sum up, TCR activates CD8^+^ T cells to induce the transfer of cellular metabolic levels to glycolysis, and the synergistic effect of CD28 leads to an upregulation of CD8^+^ T cell glycolysis levels, further supporting their subsequent proliferation and differentiation; The induction of high glycolytic activity in CD8^+^ T cells are beneficial for CD8^+^ T cells to differentiate into effector cells, but seriously impairs the survival of long-lived memory cells; For effector CD8^+^ T cells, changes in glycolysis are related in IFN- γ production, the downregulation of glycolysis levels plays an important role in the production of cytokines and immune function. Therefore, it is crucial to find a method for targeted glycolysis to restore the effector function of CD8^+^ T cells; The metabolic imbalance caused by the changes of glycolytic activity not only affects the function of CD8^+^ T cells, but also affects their effector function. The enhanced selectivity of glycolysis will further restore these functions; Glycolysis may affect partial depletion of CD8^+^ T cells through the mTOR pathway and affect IFN- γ, the occurrence of adverse effects. In addition, the balance between glycolysis and fatty acid oxidation (FAO) is related to the long-term survival of memory CD8 T cells.

### Cross-talk between CD8^+^ T cells and other immune cells in tumor microenvironment

Effector CD8^+^ T cells are thought to be a homogenous group of cytotoxic cells that produce protease granzyme B, IFN-γ and multiple subsets of CD8^+^ T cells have distinct effects and cytotoxic potential [101]. CD8^+^ T cells can be discovered in TME, where they potentially influence the antitumor response and patient outcomes. We have described the metabolism of CD8^+^ T cells in TME in the previous section, and next we will discuss the crosstalk between CD8^+^ T cells and other immune cells.

#### CD4^+^ T cell

Tumor outgrowth is controlled by CD4 and CD8 T cells, there are three phases of tumor-immunity, namely elimination, equilibrium and escape (Fig. 3A) [102]. Studies about chronic viral infection and cancer have shown that CD4 T cells are necessary for CD8 T cell function, CD4^+^ T cells are dispensable for primary expansion and cytotoxic effectors of CD8^+^ T cells [103, 104]. From single cell RNA-seq, Zander and colleagues show that the formation of effector CD8^+^ T cells is critically dependent on CD4^+^ T cell under the function of IL-21 and the pathway could be used therapeutically to enhance the killer function of CD8^+^ T cells infiltrating into the tumor [105]. As for lung adenocarcinoma, the differentiation of tumor-specific CD4^+^ T follicular helper cells under the stimulation of B cells in a neoantigen-dependent manner, which promote CD8^+^ T cell effector functions and drive anti-tumor immunity [106]. In terms of the mechanism, Ahrends et al. revealed that CD4^+^ T cells help effector CD8^+^ T cells acquire their ability that involves the downregulation of PD-1 and increased motility and migration capacities. In a similar study, CD4^+^ T cell is beneficial for the antigen-specific CD8^+^ T cells clonal expansion and IFN-γ production, too [107, 108]. Microarray analysis demonstrated that without the help of CD4^+^ T cells, CD8^+^ T cells expressed elevated the levels of inhibitory receptors such as PD1, exhibited transcriptomic exhaustion and anergy profiles change [109]. CD4^+^ T cell help the TCR repertoire. CD27 directs the expression of the Pim1 gene and the antiapoptotic Bcl-2 that promotes the survival of CD8^+^ T cells and thereby increases the function of effector and memory populations, but more TCR repertoire of responder CTLs should be explored about how to prevent immune escape of tumor cells [110, 111]. In addition, the tumor-invasive capacity of CTLs can be promoted by CD4^+^ T cell [112]. So, we can summarize that CD4^+^ T cells are significant for the differentiation, effector function, antitumor of CD8^+^ T cells in TME, and it might be important for tumor immunotherapies (Fig. 3B).

#### Dendritic cell

As the most potent professional APCs, DCs play a core role in linking innate and adaptive immune responses and in the balance of CD8 T cell immunity and tolerance to tumor antigens [113]. The functions of DCs include uptake, processing and presenting antigens to activate naive antigen-specific CD4 and CD8 T cells [114]. Activated DCs can produce IL-12, which mediate Th1 differentiation and provide essential signals for the production of resident memory CD8^+^ T cells in human and mice [115–118]. DCs can provide a friendly extracellular microenvironment for T lymphocyte activation, and DCs-derived IL-15 can promote CTL differentiation (Fig. 3B) [119, 120]. Batf3, also known as Jun dimerization protein p21SNFT, is important for DCs. Hildner and colleagues clarified that the abilities of cross-presentation and antitumor immunity were impaired in Batf3^−/−^ mice, which downregulated CD8 T cell-mediated anti-tumor immunity [121]. Broz et al. identified that CD103^+^ DCs not only induce the proliferation of naive CD8^+^ T cells, but also establish CTLs in the TME, and it’s the mediator that transport solid tumor antigens from TME to tumor draining lymph nodes for CD8^+^ T cells. The Cancer Genome Atlas (TCGA) database analysis indicated that CD103^+^/CD103^−^ is strongly correlates with cancerous patients survival [122–124]. From the study about melanoma, the activation of β-catenin signaling reduces the numbers of intratumoral CD103^+^ DCs that prevent tumor-specific T cell priming and anti-tumor immunity, at the same time, CD103^+^ DCs are also critical target for the efficacy of immunotherapy with PD-L1 and Braf inhibition [123, 125]. The number of tumor infiltrated CD8^+^ CD103^+^ T_RM_ cells have been identified correlating with prolonged survival and better prognosis in ovarian, endometrial, breast and lung cancer [126–130]. It’s worth noting that Spranger et al. have shown that vaccination with DCs improved the efficacy of anti-PD-L1 and anti-CTLA-4 immunotherapy [125]. To sum up, we need to further explore the role of DCs and subsets on other T cells, and further clarify how, when and where DCs present tumor antigens to interact with CD8^+^ T cells. DCs offer an opportunity to manipulate CD8^+^ T cells and vaccine to generate anti-tumor immunity in the TME, it will be a promising target for tumor therapy.

#### Macrophages

Macrophages are highly multifunctional and plastic cells that participate in tissue development, homeostasis, clearance of cellular debris, elimination of pathogens, regulation of inflammatory responses and tumors. It is generally simplified into two categories: M1 or M2 macrophages (Fig. 3B) [131, 132]. Generally speaking, M1 macrophages have the anti-tumor roles, and M2 macrophages promote the occurrence and development of tumors. Specifically, M1 macrophages have two different effects, one is directly mediate cytotoxicity to kill tumor cells and another is antibody dependent cell mediated cytotoxicity (ADCC), which is faster than cytotoxicity [133]. Tumor-associated macrophages (TAMs) participate in the regulation of the TME. TAMs are widely present in various tumors, secreting a variety of cytokines such as epithelial growth factor (EGF), platelet-derived growth factor (PDGF), TGF, hepatocyte growth factor (HGF), and epithelial growth factor receptor (EGFR) family, and correlate with tumor growth, invasion, metastasis, and treatment-ineffectiveness [134–136]. Studies revealed that Treg cells can depress IFN-γ secreted by CD8^+^ T cells to promote the polarization of M2-like TAMs [137]. Intravital imaging studies shown that antigen-specific CD8^+^ T cells preferentially localize in TAM-rich areas in the TME [122, 138, 139].

TAMs negatively regulate T cell activation and hinder CD8^+^ T cell reaching tumor cells that limit the efficacy of anti-PD-1 treatment. Combinational treatment of anti-PD-1 with PLX3397, an inhibitor of colony-stimulating factor-1 receptor (CSF-1R), increases the accumulation of CD8^+^ T cells in malignant cells and delays tumor progression [139]. Zhang et al. reported that IL-15Rc/HIF-1α/CX3CL1 signal pathway serves as a crosstalk between macrophages and CD8^+^ T cells. IL-15Rα^+^ TAMs reduce the levels of CX3CL1 to reduce CD8^+^ T recruitment through releasing the IL-15/IL-15Rα complex in the TME [140]. The expression of IL-10 by macrophages depresses IL-12, which is produced by intratumoral DCs that suppress CD8^+^ T cells and response to paclitaxel and carboplatin, while, IL-10R blockade increase the expression of IL-12 and improve the treatment outcomes [115]. In the same experiment, Petty and colleges reported that hedgehog signal is critical for TAM M2 polarization and tumor growth that suppresses CD8^+^ T cell recruitment to the TME through the inhibition of CXCL9 and CXCL10 production [141]. In contrast, FOLR2^+^ tissue-resident macrophages are positively correlated with tumor immunity, efficiently prime effector CD8^+^ T cells, and better patient survival [142]. These studies highlight specific roles for TMEs and its subsets for targeted therapeutic interventions in macrophages-based cancer therapies, and macrophages could be a promising aim for immune therapy.

The composition and function of TME are also undergoing dynamic changes during the development of cancer. Through this review, we recognize the enormous complexity and interconnectedness of TME, as well as its diversity in different organs and patients. Targeted therapy of cells, biological processes, and signaling pathways in TME are considered promising strategies that can be extended to all types of cancer. The large number of co-immune and stromal cells found in TME are genetically stable, making them easier to target compared to cancer cells with unstable genomes [143, 144]. For example, standard treatments including chemotherapy and radiation therapy can cause changes in TME, regulating its therapeutic effect in an external manner to cancer cells, enhancing or interfering with the response. It is worth noting that adaptability and intrinsic resistance may be obstacles to targeted treatment of TME. Despite these challenges, there is great hope for expanding treatment strategies targeting TME, including depletion or "reprogramming" of cancer promoting host cells in TME; Intervention measures to modify extracellular matrix (ECM), matrix components, and extracellular vesicles (EVs); Cell based therapies and vaccines; And immune checkpoint inhibitors. Moreover, integrating multiple cancer model data and advanced computational analysis, including artificial intelligence, has the potential to adopt a comprehensive system level approach about analyzing and integrating all the complexities of TME to identify key nodes [145]. In addition, significant advances in bioengineering will provide a platform for large-scale testing, such as in ex vivo organoids and tissue slices that accurately recurrent organ specific TMEs [146–148].

## Conclusions and prospective

By RNA sequencing (RNA-seq) and transposase-accessible chromatin sequencing (ATAC-seq), Pritykin defined a dysfunction and underlying transcriptional drivers and revealed a state of functional and dysfunctional T cell across cancer and infection models [149]. Zheng et al. analyzed T cell populations from hepatocellular carcinoma (HCC), and revealed distinct subtypes and clonal expansion of infiltrating lymphocytes [150]. A better understanding of CD8^+^ T cell clustering, dynamic, markers and developmental trajectory will provide more therapeutic strategies for diseases. In this review, we discuss CD8^+^ T cells development, metabolism and interaction with tumor microenvironment.

In past few years, novel checkpoint blockades have given some attention in the treatment of multiple solid cancers. Antibodies targeting inhibitory receptors including PD-1 successfully increase T cell function and clinical efficacy in tumors. As summarized in Table 4, the treatment of PD-1 combined with chemotherapy, radiotherapy, targeted therapy and cytokines make a promising outcome [151, 152]. It is amazing that cancer vaccines have also attracted lots of interest, and Sipuleucel-T and T-VEC have been approved by FDA [153, 154]. However, cancer vaccines have not shown desired results in several clinical trials, mainly because that cancer vaccines can’t effectively active T cell and the safety of the vaccine deserves further investigation. The reasons of CD8^+^ T cells exhaustion have been explored, and we need to clarify more biomarkers to predict CD8^+^ T cells exhaustion and status, the most meaningful question is whether and how the exhaustion of CD8^+^ T cells can be reversed. Additional combinatorial strategies should be considered, for example, antiangiogenic inhibitors targetingvascular endothelial growth factor (VEGF), which can promote infiltration of immunostimulatory cells, block immunosuppressive effects in the TME, and improve drug delivery [155]. Recombination of clinically approved treatment methods or conversion of “cold tumor” to “hot tumor” through different drug administration is an inspiring goal. Together, existing and future approaches are helpful for understanding CD8^+^ T cells, and we should be optimistic about therapy that will be applied to human diseases.

**Table 4:** In *vivo* experiments related to CD8^+^ T cells

Disease	Treatment	Results	Limitations	Refs.
Glioblastoma	Neoantigen vaccine	Designed a neoantigen-personalized tumor vaccine for glioblastoma patients, which successfully promoted the anti-tumor response of CD8^+^ T cells	Limited for patients who did not receive dexamethasone during vaccine priming; post vaccination, T cells expressed multiple co-inhibitory receptors	[188]
NSCLC	Neoadjuvant therapy	lymph node metastases, cancer microvessels and cancer‐associated fibroblasts promote CD8^+^ T cell exclusion and dysfunction	Limited cohort; uncertain whether the setting of a positive threshold during the image analysis; lack of functional tests on T cells and other immune cells	[189]
NSCLC	Anti PD-1 treatment	CD103^+^CD8^+^ infiltrating lymphocytes could serve as a predictive biomarker for PD-1 based immunotherapy	Uncertain CD103^+^CD8^+^ TILs are enriched for tumor antigen specific CTL	[190]
NSCLC	ICB	find a relationship between self-renewing CD8^+^ T cells and response of cancer patients to PD-1 blockade	Cannot exclude the intrinsic difference in patients	[191]
NSCLC	Bevacizumab combined with anti PD-1 treatment	improved abnormal tumor vessels and enhanced T lymphocytes cytotoxic and prolong patient’s survival time function of CD8^+^ T cells in lung cancer	Unknown how to integrate VEGF/VEGFR inhibitors combine with ICIs and the mechanisms needed to elucidate	[192]
NSCLC	4-1BB agonism combining with anti PD-L1	anti PD-L1 combine with 4-1BB induced further tumor regression and enhanced survival in tumor-bearing mice	Need make deeper characterizations of the CD103^+/−^ CD8^+^ T cells beyond immune molecules	[193]
NSCLC	ICB	CD28 is advocated as a key determinant in CD8 T cells and provides feasible biomarkers of ICB response	CD137 and ICOS failed to provide functional advantage to CD28^−^ T cells in the tumor site; cannot rule out that a fraction of intra-tumor PD1^+^CD28^−^ T cells	[194]
NSCLC	anti PD-L1 treatment	identified a heterogeneous population of neoantigen-specific CD8^+^ T cells with a late effector-like phenotype	limited pre- and post-treatment patient samples	[195]
ESCC	NICB	CD8^+^ Tex-SPRY1 cells predict response, interactions with macrophages and B cells, enhance ICB response and improved survival for ICB therapy	Unclear whether the regulation of progenitor cell like CD8^+^ Tex cells interacting with other immune cells contributes to the immunotherapy response	[196]
Cervical cancer	HPV E6/E7-targeted therapeutic vaccination combined with radiotherapy	CD103 is a biomarker for tumor-reactive T cell infiltration of cervical cancers and E6/E7-targeted immunotherapy	Unknown the precise differentiation status of CD8 coexpression in cervical cancer	[197]
Melanoma	pembrolizumab	Tumor-resident CD8^+^ T-cell numbers are more prognostic than total CD8^+^ T cells	Absence of information on subsets that can not determine whether the protective response was associated with any particular subset	[198]
Melanoma	BzATP	P2RX7 stimulation is a novel therapeutic treatment to enhance tumor immunotherapy	unclear what role P2RX7 would play in adoptive immunotherapy by CD8^+^ T cells; need for careful evaluation of how tumor-specific T cells are activated in order to address whether P2RX7 plays a beneficial role	[199
BC	ICB	CD8^+^ T_RM_ cells contribute to BC immuno- surveillance and are the key targets of modulation by immune checkpoint inhibition	Unclear the direct or indirect mechanisms in vivo	[200]
Melanoma	Activin-A	activin-A offer new therapeutic opportunities to overcome CD8^+^ T cell exclusion and immunotherapy resistance	Limited bioavailability in Tregs or CD4^+^ T cell; unclear the potential roles of DCs and of monocyte recruitment by CCR4 ligands	[201]
CRC	radical-intent resection of the primary tumor	CD8^+^ MeTIL markers could measure CD8^+^ TILs distributions	Low proportion of stage IV disease in this cohorts, and more metastatic tumors are needed	[202]
BC	radical cystectomy and adjuvant chemotherapy	TIGIT^+^ CD8^+^ T-cells were associated with suppressive immune contexture and it can regard as a biomarker for treatment	Unknown the mechanism of TIGIT shaping the dysfunction state of CD8^+^ T cells and the synergistic effect of double immune checkpoint blockade in BC were worthy of further study	[203]
HCC	δ-Catenin peptide vaccines	active CTLs, enhance the infiltration of CD8^+^ T cells into tumors and enhance the secretion of IFN-γ	Didn’t check the therapy effects ofδ-Catenin peptide vaccines combined with anti-CTLA-4 or anti-PD-1 mAbs	[204]

*NSCLC* non-small cell lung cancer, *ICB* immune checkpoint blockade, *ICI* immune checkpoint inhibitors, *ESCC* esophageal squamous cell carcinoma, *NICB* neoadjuvant immunotherapy, *BC* breast cancer, *CRC* colorectal cancer, *BC* bladder cancer, *HCC* hepatocellular carcinoma. *CTLs* cytotoxic T lymphocytes

## Data Availability

All data are available in the main text.

## Abbreviations

MHC-I: Major histocompatibility complex
APCs: Antigen presenting cells
TNF: Tumor necrosis factor
PD-1: Programmed death receptor 1
PD-L1: Programmed cell death 1 ligand 1
Eomes: Eomesodermin
mTOR: Mammalian target of rapamycin
TCR: T cell receptor
IFN: Interferon
DCs: Dendritic cells
LCMV: Lymphocytic choriomeningitis virus
TGF-β: Transforming growth factor β
AMPK: AMP-dependent protein kinase
HREs: Hypoxia response elements
GLUT1: Glucose transporter 1
CTLs: Cytotoxic T lymphocytes
TME: Tumor microenvironment
LAG3: Lymphocyte-activation gene 3
Tim-3: T cell immunoglobulin domain and mucin domain 3
TIGIT: Ig and ITIM domain
CTLs: Cytotoxic T lymphocytes
TAMs: Tumor-associated macrophages
HCC: Hepatocellular carcinoma
NK: Natural killer cells
SLECs: Short-lived effector cells
TEs: Terminal effector cells
MPs: Memory precursors
TILs: Tumor-infiltrating lymphocytes
FAO: Fatty acid oxidation
EVs: Extracellular vesicles
ECM: Extracellular matrix
VEGF: Vascular endothelial growth factor
